# Efficacy and Safety of IncobotulinumtoxinA in Older Patients with Upper Limb Spasticity: A Pooled Analysis

**DOI:** 10.3390/geriatrics10060155

**Published:** 2025-11-24

**Authors:** Michael C. Munin, Alexandre Camões-Barbosa, Carlos Cordero-García, Alessio Baricich, Stefano Carda, Michael Althaus, Georg Comes, Matteo Vacchelli, Jörg Wissel

**Affiliations:** 1 Department of Physical Medicine and Rehabilitation, University of Pittsburgh School of Medicine, Pittsburgh, PA 15213, USA; muninmc@upmc.edu; 2 Neurotoxin Clinic Neurophysiology Unit, Unidade Local de Saúde de S. José, 1150-199 Lisbon, Portugal; alexandre.camoes@gmail.com; 3 Department of Physical Medicine and Rehabilitation, Juan Ramón Jiménez University Hospital, 21005 Huelva, Spain; carlos.cordero.sspa@juntadeandalucia.es; 4 Department of Biomedical Sciences, Humanitas University, Pieve Emanuele, 20072 Milan, Italy; alessio.baricich@med.uniupo.it; 5 Rehabilitation Unit, IRCCS Humanitas Research Hospital, Rozzano, 20089 Milan, Italy; 6 Neuropsychology & Neurorehabilitation, Lausanne University Hospital (CHUV), 1005 Lausanne, Switzerland; stefano.carda@gmail.com; 7 Merz Therapeutics GmbH, 60318 Frankfurt am Main, Germany; michael.althaus@merz.de (M.A.); georg.comes@merz.de (G.C.); 8 Neurology and Psychosomatic at Wittenbergplatz, 10787 Berlin, Germany; joerg@schwarz-wissel.de; 9 Brandenburg Health Campus, University of Potsdam, 14469 Potsdam, Germany

**Keywords:** aged, botulinum toxins, incobotulinumtoxinA, muscle spasticity, safety, stroke, treatment outcome

## Abstract

**Background/Objectives**: The aim of this study was to compare the efficacy and safety of a single cycle of incobotulinumtoxinA versus placebo in pooled data from older patients (aged ≥65 years) with upper limb spasticity (ULS). **Methods**: This study was a post hoc analysis of pooled data from seven prospective, multicenter, phase II or III trials of incobotulinumtoxinA in adult patients aged ≥65 years from across the world with post-stroke ULS or upper and lower limb spasticity, including a subgroup with moderate-to-severe ULS. Changes from baseline in ULS severity were evaluated using the (modified) Ashworth Scale across different spasticity patterns at 4 and 12 weeks after incobotulinumtoxinA injection. **Results**: In 267 older patients with ULS, including a subgroup of 207 with moderate-to-severe ULS, all ULS patterns statistically analyzed (elbow flexion, thumb-in-palm, clenched fist, wrist flexion, and pronated forearm) were improved more by incobotulinumtoxinA than placebo at week 4 (*p* < 0.05). For most of these patterns, the difference remained significant at week 12 (*p* < 0.05). IncobotulinumtoxinA was generally well tolerated. **Conclusions**: This study, which analyzed data from the largest cohort of older patients in the literature, provides information regarding the use of incobotulinumtoxinA in ULS, the efficacy and favorable safety profile of incobotulinumtoxinA for the treatment of ULS in older patients, particularly in those with moderate-to-severe spasticity, was confirmed.

## 1. Introduction

More than 12.2 million new strokes occur each year globally, with the vast majority in individuals aged ≥50 years and 38% in individuals aged ≥70 years [1]. In 2010, 69% of strokes occurred in those aged ≥65 years [2]. A recent meta-analysis that included data from 36 studies from around the world reported an estimated overall prevalence of stroke in the elderly of 7.4%, with an increasing prevalence with rising age, and the highest prevalence in the USA (9.4%) [3].

In care facilities for elderly people, spasticity is common, particularly after stroke, and is often undertreated [4]. For example, in individual studies of residents of care facilities for elderly people, 70% with a history of stroke had upper-limb spasticity (ULS) [5], and 22% had spasticity, irrespective of a history of stroke [6]; in the latter study, only 11% of those identified with spasticity had a prior diagnosis of spasticity and were receiving treatment [6]. In another study, more than three-quarters of those with spasticity had related needs, most of which were unmet (76%) [7].

Spasticity has several unwanted consequences. It is significantly associated with the development of stretch/abnormal muscle contraction-induced pain [8] and pressure ulcers, including in areas such as the elbows and palms, in the elderly who are bedridden (adjusted odds ratio of 11; *p* < 0.05) [9]. Post-stroke spasticity and its manifestations can also affect recovery after stroke [10], but motor and functional outcomes can improve if patients receive early diagnosis and appropriate treatment [11].

Botulinum toxin is a first-line treatment for post-stroke spasticity when used in combination with appropriate physical therapy, other anti-spasticity strategies and postural management programs (which aim to provide a planned approach encompassing all activities and interventions that affect posture and function, such as seating, beds, and walking frames, as well as ensuring that the patient is able to change position and obtain optimal positioning of their head, body, and limbs) [12,13]. It is recommended for this use in many guidelines [13,14,15]. However, elderly patients are often under-represented in clinical trials for numerous reasons such as inclusion and exclusion criteria and institutional and logistical issues [16,17]. Data from clinical trials of botulinum toxin in ULS specific to this patient population are scarce, although one case series showed that reducing spasticity with botulinum toxin A assisted in the healing of long-standing treatment-resistant hand ulcers in nursing home residents [18]. In addition, the geriatric population is arbitrarily defined. The most common definition used in medicine appears to be that of the European Medicines Agency, which uses a definition of patients aged ≥65 years but emphasizes the importance of including those aged ≥75 years when possible [19].

The aim of this study was to compare the efficacy and safety of a single cycle of incobotulinumtoxinA versus placebo in pooled data from a large sample of older patients (aged ≥65 years), including those with moderate-to-severe ULS, enrolled in clinical trials.

## 2. Materials and Methods

This post hoc analysis evaluated changes from baseline in ULS severity across different spasticity patterns at 4 and 12 weeks after incobotulinumtoxinA injection. It used patient-level data from seven prospective, multicenter, phase II or III studies of incobotulinumtoxinA (Xeomin^®^; Merz Pharmaceuticals GmbH, Frankfurt, Germany) in the treatment of upper [20,21,22,23,24] (Merz Therapeutics GmbH, Frankfurt/Main, Germany. Internal document MRZ 60201–0307, 2008) or upper and lower [25] limb muscle spasticity in adults from across the world and with a range of ethnicities (Table 1). All ULS phase II/III studies conducted by the study sponsor (Merz Therapeutics GmbH), including two unpublished studies that were prematurely completed because of inadequate recruitment [24], were used in this analysis to ensure sufficient data availability to conduct the pooled analyses. Methodological details and results of the remaining five studies have been published [20,21,22,23,25].

Of the seven studies, five were double blind, randomized, and placebo-controlled, whereas two evaluated different incobotulinumtoxinA doses or dilutions and were not placebo-controlled (Table 1). In all studies, patients aged ≥18 years who had not received botulinum toxin A injections within at least 4 months of screening received incobotulinumtoxinA injections as appropriate for their condition. Each injection was followed by at least 12 weeks of observation and assessment. In all but one study, patients could receive their first cycle of incobotulinumtoxinA at a total intended body dose of up to 400 U; in the remaining study, the maximum dose was 200 U split across the wrist and elbow flexors. In some studies, patients could receive multiple doses of incobotulinumtoxinA; however, the current analyses are concerned only with the first injection cycle. The mean (standard deviation [SD]) age of each population in published studies ranged from 53.7 (SD 13.1) to 59.7 (SD 11.7) years [20,21,22,23,25]. The focus of these analyses is patients aged ≥65 years with ULS manifesting as a variety of spasticity patterns.

All studies were conducted in accordance with the Declaration of Helsinki and good clinical practice and were approved by the ethics committee for each participating site. Before study participation, all patients provided written informed consent.

### 2.1. Overall Analyses

Patient-level data from the seven studies of incobotulinumtoxinA in ULS [20,21,22,23,24,25] were pooled from patients with mild, moderate, and severe ULS. Baseline characteristics of included participants were descriptively reported as mean (SD) or number (%) of patients.

The differences between incobotulinumtoxinA and placebo in the least squares mean change from the baseline in the Ashworth Scale (AS) score at 4 and 12 weeks post-injection were calculated based on spasticity pattern: elbow flexion, thumb-in-palm, clenched fist, wrist flexion, shoulder (internally rotated/extended/adducted), and pronated forearm. Two studies used modified AS (mAS) scores [23,24], and these data were included with the AS score data ([m]AS scores). Reported data were evaluated using analysis of covariance with sex, baseline disease severity (mild, moderate, and severe), time since first diagnosis of spasticity (0–2, 3–5, 6–10, and >10 years), and baseline (m)AS score as covariates.

Safety was also assessed throughout the 12-week analysis period.

### 2.2. Subgroup Analysis: Patients with Moderate-to-Severe ULS

Spasticity severity was determined by baseline AS score using a top-down procedure to distinguish between the categories. Severe ULS was defined as an AS score of 4 in at least two assessed joints; once these patients were categorized, the remaining patients were categorized as having moderate ULS if they had an AS score ≥ 3 in at least two assessed joints (i.e., one joint with a score of 4 and at least one joint with a score of 3, or at least two joints with a score of 3 and not severe ULS) or mild ULS (not moderate or severe). Two studies used mAS scores [23,24].

Patient-level data from patients with moderate-to-severe ULS were extracted and pooled from six of the above studies [20,21,22,23,25]. One of the seven studies was conducted in patients with an acute cerebrovascular event to determine the effects of incobotulinumtoxinA on the development of ULS and included no patients with moderate-to-severe ULS [24].

Baseline characteristics of patients with moderate-to-severe ULS were descriptively reported as mean (SD) or number (%) of patients.

Differences between incobotulinumtoxinA and placebo in the least squares mean change from baseline in the (m)AS score at 4 and 12 weeks post injection were calculated. Reported data were analyzed using analysis of covariance, as per the main analyses.

Safety was also evaluated throughout the 12-week period in this subgroup of patients.

## 3. Results

Mean (SD) age of the 267 older patients with mild, moderate, or severe post-stroke ULS was 70.3 (4.2) years, with 15.0% aged ≥75 years (Table 2). Overall, 37.5% of patients were female. The etiology of spasticity was stroke in >99% of patients, and the mean (SD) time from spasticity diagnosis was 5.0 (5.4) years. Baseline characteristics were generally similar between the incobotulinumtoxinA- and placebo-treated patients, although there was a slight preponderance of females (34.5% vs. 46.9%) and the mean (SD) time since diagnosis of spasticity was slightly shorter (5.2 [5.5] vs. 4.3 [4.8] years) in the placebo group.

**Figure 1:**
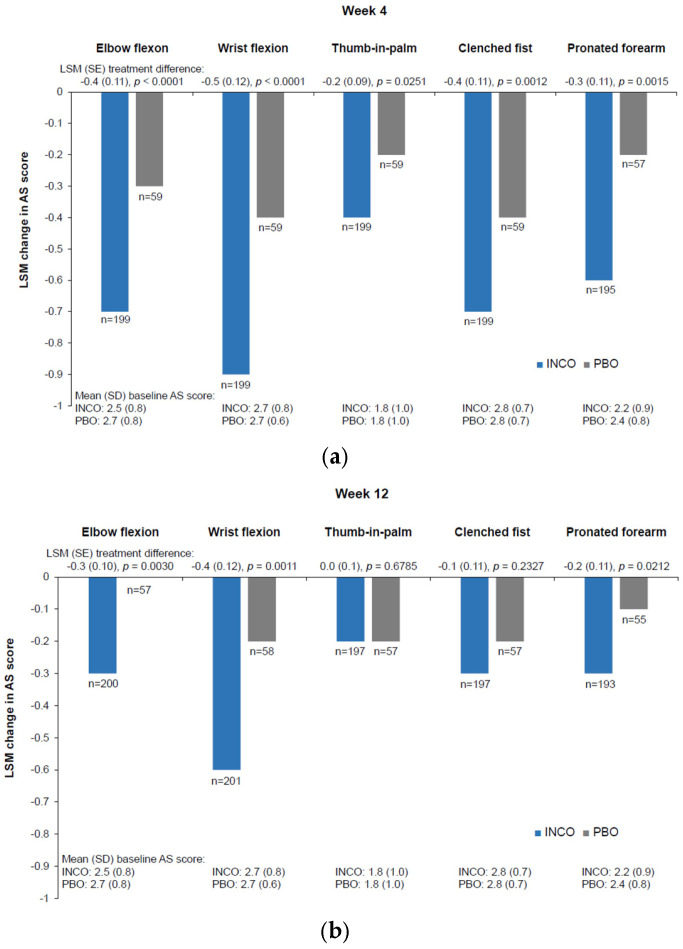
Change from baseline in AS score ^a^ (**a**) at week 4 and (**b**) at week 12 after injection of incobotulinumtoxinA or placebo in older patients (aged ≥65 years) with mild, moderate, or severe ULS by spasticity pattern; as observed analyses. ^a^ One of the pooled studies used the modified AS. AS: Ashworth Scale; INCO: incobotulinumtoxinA; LSM: least squares mean; PBO: placebo; SD: standard deviation; SE: standard error; ULS: upper limb spasticity.

For all ULS patterns with sufficient power for statistical analysis (elbow flexion, thumb-in-palm, clenched fist, wrist flexion, and pronated forearm), incobotulinumtoxinA produced greater improvement in (m)AS score than did placebo at week 4 (*p* < 0.05) (Figure 1). For most of these patterns (elbow flexion, wrist flexion, and pronated forearm), the difference remained significant at week 12 (*p* < 0.05) (Figure 1). IncobotulinumtoxinA was associated with improvements in all ULS patterns from baseline at both week 4 and week 12 (Figure 1). However, the ULS shoulder pattern had insufficient power to allow comparison between treatments, as only 41 incobotulinumtoxinA and two placebo recipients were treated in this pattern.

**Figure A1:**
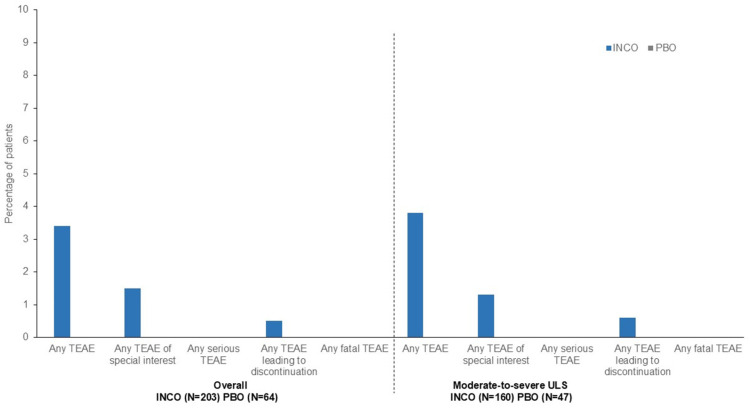
Pooled safety data for incobotulinumtoxinA and placebo in older patients (aged ≥65 years) with upper limb spasticity: treatment-related treatment-emergent adverse events. INCO: incobotulinumtoxinA; PBO: placebo; TEAE: treatment-emergent adverse events; ULS: upper limb spasticity.

**Figure A2:**
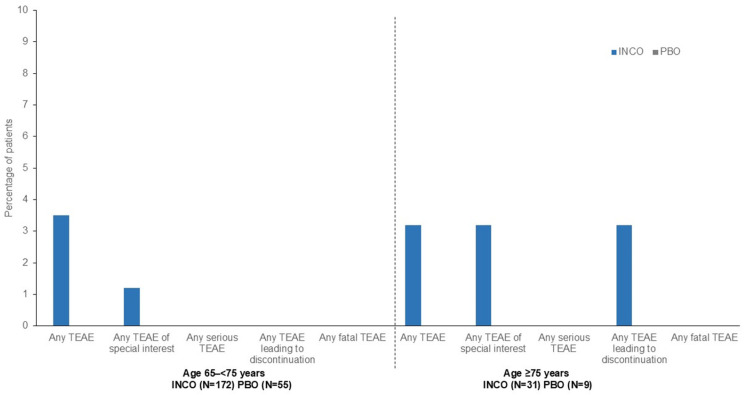
Pooled safety data for incobotulinumtoxinA and placebo in older patients (aged ≥65 years) with upper limb spasticity by age group: treatment-related treatment-emergent adverse events. INCO: incobotulinumtoxinA; PBO: placebo; TEAE: treatment-emergent adverse events; ULS: upper limb spasticity.

IncobotulinumtoxinA was generally well tolerated, with no treatment-related serious adverse events or deaths reported (Table 3; Appendix Figure A1), and with few differences reported between patients aged 65 to <75 years and those aged ≥75 years (Table 4; Appendix Figure A2). The administered incobotulinumtoxinA doses were 95–400 U in those aged 65 to <75 years and 75–400 U in those aged ≥75 years.

Mean (SD) age of the 207 older patients with moderate-to-severe ULS was 70.0 (4.1) years, and 13.5% were aged ≥75 years (Table 5). Mean (SD) time from spasticity diagnosis was 5.3 (5.3) years in this subgroup, and, overall, 85.5% of these patients had moderate and 14.5% had severe ULS.

**Figure 2:**
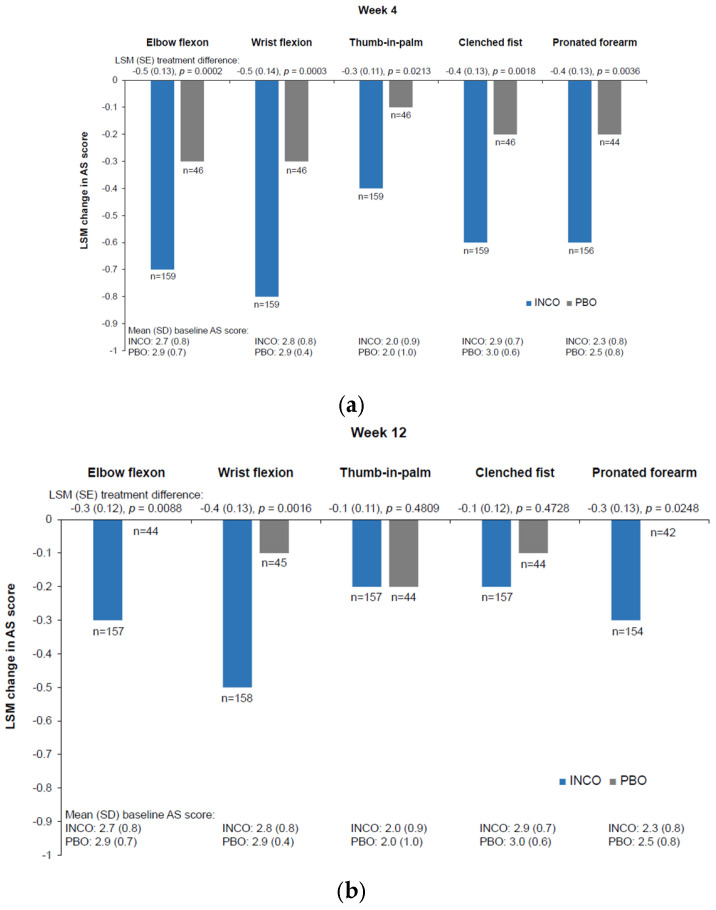
Change from baseline in AS score ^a^ (**a**) at week 4 and (**b**) at week 12 after injection of incobotulinumtoxinA or placebo in older patients (aged ≥65 years) with moderate-to-severe ULS by spasticity pattern; as observed analyses. ^a^ One of the pooled studies used the modified AS. AS: Ashworth Scale; INCO: incobotulinumtoxinA; LSM: least squares mean; PBO: placebo; SD: standard deviation; SE: standard error; ULS: upper limb spasticity.

For all ULS patterns with sufficient power for statistical analysis (elbow flexion, thumb-in-palm, clenched fist, wrist flexion, and pronated forearm), incobotulinumtoxinA produced greater improvement in AS score than did placebo at week 4 (*p* < 0.05) (Figure 2). For most of these patterns (elbow flexion, wrist flexion, and pronated forearm), the difference remained significant at week 12 (*p* < 0.05) (Figure 2). IncobotulinumtoxinA was associated with improvements in all ULS patterns from baseline at both week 4 and week 12 (Figure 2). However, the ULS shoulder pattern had insufficient power to allow comparison between treatments, as only 37 incobotulinumtoxinA and two placebo recipients were treated in this pattern.

## 4. Discussion

Elderly patients are often under-represented in clinical trials [16,17], and the current analyses were designed to help address this issue. In a large group of older patients with post-stroke ULS (aged ≥65 years), including a subgroup with moderate-to-severe ULS, from around the world, this study showed that incobotulinumtoxinA improved upper limb muscle tone and was well tolerated after a single injection cycle. The study population had a mean (SD) age of 70.3 (4.2) years, with 15.0% aged ≥75 years. This contrasts with the mean (SD) age of each population in the published studies of 53.7 (13.1) to 59.7 (11.7) years [20,21,22,23,25]. International regulatory guidelines encourage studies of drugs that will be beneficial for elderly patients in geriatric populations [19].

In both the overall cohort of older patients with post-stroke ULS and the subgroup with moderate-to-severe ULS, incobotulinumtoxinA significantly reduced (improved) (m)AS scores at week 4, to a greater degree than the placebo, across all the ULS patterns analyzed. Importantly, these significant improvements compared with placebo were maintained for a number of ULS patterns at week 12, with all patterns continuing to show improvement versus baseline at this time point. These results build on and are in line with those of the individual studies from which the patient data were obtained [20,21,22,23,25]. These studies showed that treatment with incobotulinumtoxinA improved muscle tone across ULS patterns and reduced spasticity-associated pain and disability [20,21,22,23], allowing many patients to achieve their treatment goals [25]. The current findings are supported by the encouraging results of studies in upper limb muscle overactivity in elderly populations. A small pilot study has investigated incobotulinumtoxinA in the treatment of upper limb paratonia in an elderly population with dementia and revealed promising results in terms of both efficacy and safety [26]. Similarly, a retrospective single-center study of 49 patients aged >70 years with spastic hypertonia and/or a dystonia found that more than half the patients had improved quality of life, functional status, and/or pain after treatment with botulinum toxin [27].

The tolerability profile of incobotulinumtoxinA in older patients was also in line with that reported in adult patients aged ≥18 years, particularly when adverse events of special interest (AESI) and treatment-related treatment-emergent adverse events were considered [20,21]; in one trial, the incidence of treatment-related treatment-emergent adverse events and AESI appeared higher than that reported for this population of older patients [25]. In these trials, AESI were defined based on a prespecified list of adverse events that could potentially indicate toxin spread and included dyspnea, dry mouth, dysphagia, speech problems, and muscle weakness [20,21,25].

The current results must be considered in light of recent findings on underutilization of botulinum toxin treatment in patients with post-stroke ULS [28]. Notably, use of botulinum toxin therapy declined with increasing age at the time of stroke, with only 3.8% of patients aged 60–69 years, 1.8% of those aged 70–79 years, 0.5% of those aged 80–89 years, and 0.1% of those aged ≥90 years receiving this treatment. It is possible that a lack of data concerning the use of botulinum toxin in the elderly contributed to this finding. However, the appropriate use of botulinum toxin therapy in survivors of stroke is important given the substantial detrimental impact of spasticity on their quality of life, as reported by numerous studies [29,30,31,32], and this applies to survivors of all ages.

Strengths of these analyses include the wide international geographic catchment of the included studies; the use of a widely accepted measure of muscle tone, the (m)AS; that most of the studies were double blind, randomized, and placebo-controlled for the evaluated injection cycle; and that individual study results were not combined (rather, findings were calculated based on patient-level data). However, there was potential for individual studies to influence our findings because of the contribution of different numbers of patients to the pooled analyses. Additionally, the contributing studies were not all in the same drug development phase and their designs differed. These design variations included the use of different assessment scales, which prevented the investigation of how the improvements in (m)AS scores translated to activities of daily living and carer burden. No analyses were performed to investigate potential differences in baseline patient characteristics between studies, which may also have impacted our findings. Finally, comprehensive searches for studies that could contribute to the analyses were not performed; instead, all studies were identified by their sponsor—Merz Therapeutics GmbH—which ensured sufficient data availability.

## 5. Conclusions

This is the largest cohort of older patients to provide data regarding the use of incobotulinumtoxinA in ULS. Overall, results of this pooled analysis support and extend the efficacy and favorable safety profile of incobotulinumtoxinA for the treatment of ULS in older patients, particularly in those with moderate-to-severe spasticity.

## Figures and Tables

**Table 1:** Summary of prospective clinical trials included in the pooled analysis of incobotulinumtoxinA in older patients (aged ≥65 years) with upper limb spasticity.

Reference	Study Design	Treatments in First Injection Cycle ^a^	Older Patients ^b^, n	Baseline Patient ^b^ Characteristics (Study Location)	Duration ^c^
MRZ_60201_0307 (unpublished) ^d^	Double blind, randomized, placebo-controlled, parallel group, multicenter, phase II pilot	INCO ≤ 400 U Placebo	4 2	33.3% female; mean age 70.2 years; 100.0% white; time since spasticity diagnosis 1.2 years (Germany)	12 weeks
NCT00432666 [20]	Double blind, randomized, placebo-controlled, multicenter, phase III	INCO ≤ 400 U (intended) Placebo	22 16	39.5% female; mean age 69.5 years; 100.0% white; time since spasticity diagnosis 5.6 years (Czech Republic, Hungary, Poland)	≤20 weeks
NCT01392300 [21]	Double blind, randomized, placebo-controlled, multicenter	INCO 400 U Placebo	48 32	43.8% female; mean age 69.6 years; 88.8% white, 7.5% Asian, 2.5% Black; time since spasticity diagnosis 3.3 years (Czech Republic, Germany, Hungary, India, Poland, Russian Federation, USA)	12 weeks
NCT00465738 [22]	Observer blind, randomized, parallel group, multicenter	INCO (20 or 50 U/mL) ≤400 U (median 300 U)	58	44.8% female; mean age 71.4 years; 84.5% White; time since spasticity diagnosis 5.7 years (Austria, France, Germany, Italy, Portugal, Spain, Switzerland, UK)	20 weeks
NCT01603459 [25]	Open label, non-randomized, single arm, multicenter	INCO 400 U	40	20.0% female; mean age 69.5 years; 85.0% White, 2.5% Asian; time since spasticity diagnosis 5.6 years (Canada, France, Germany, Italy, Norway, Portugal, Spain, USA)	36–48 weeks
JapicCTI-153029 [23]	Double blind, randomized, placebo-controlled, multicenter, phase III	INCO 250 U INCO 400 U Placebo	28 11	30.8% female; mean age 70.8 years; 100.0% Asian; time since spasticity diagnosis 7.8 years (Japan)	12 weeks
MRZ_60201_0528 [24] (unpublished) ^d^	Double blind, randomized, placebo-controlled, multicenter, phase II	INCO 100–200 U Placebo	3 3	33.3% female; mean age 75.0 years; 100.0% white; time since spasticity diagnosis 0.0 years (Germany)	12 weeks

^a^ Most of these studies evaluated multiple treatment cycles, which may have included different doses; however, the current analyses are concerned only with the first treatment cycle. ^b^ Patients from the study included in the current analyses. ^c^ Duration of the first injection cycle. ^d^ These two unpublished studies were terminated early because of low recruitment. INCO: incobotulinumtoxinA; UK: United Kingdom; USA: United States of America.

**Table 2:** Baseline characteristics of older patients (aged ≥65 years) with upper limb spasticity who received a single dose of incobotulinumtoxinA or placebo.

Parameter	IncobotulinumtoxinA (n = 203)	Placebo (n = 64)	Total (n = 267)
Mean (SD) age, years	70.3 (4.2)	70.2 (4.2)	70.3 (4.2)
Age ≥ 75 years, n (%)	31 (15.3)	9 (14.1)	40 (15.0)
Female, n (%)	70 (34.5)	30 (46.9)	100 (37.5)
Race, n (%)			
White	154 (75.9)	50 (78.1)	204 (76.4)
Black or African American	1 (0.5)	1 (1.6)	2 (0.8)
Asian	33 (16.3)	13 (20.3)	46 (17.2)
Other/missing	15 (7.4)	0	15 (5.6)
Mean (SD) weight, kg	74.2 (13.6)	71.7 (13.1)	73.6 (13.5)
Baseline disease severity, n (%)			
Mild	43 (21.2)	17 (26.6)	60 (22.5)
Moderate	138 (68.0)	39 (60.9)	177 (66.3)
Severe	22 (10.8)	8 (12.5)	30 (11.2)
Mean (SD) time since spasticity diagnosis, years	5.2 (5.5)	4.3 (4.8)	5.0 (5.4)
Etiology of spasticity, n (%)			
Stroke—ischemic	111 (54.7)	46 (71.9)	157 (58.8)
Stroke—hemorrhagic	27 (13.3)	16 (25.0)	43 (16.1)
Stroke—not known	63 (31.0)	2 (3.1)	65 (24.3)
Other	2 (1.0)	0	2 (0.8)
Any concomitant medication, n (%)	202 (99.5)	64 (100.0)	266 (99.6)

SD: standard deviation.

**Table 3:** Pooled safety data for incobotulinumtoxinA and placebo in older patients (aged ≥65 years) with upper limb spasticity.

Number (%) of Patients with:	Overall			Moderate-to-Severe ULS		
	INCO (n = 203)	PBO (n = 64)	Total (n = 267)	INCO (n = 160)	PBO (n = 47)	Total (n = 207)
Any TEAE	72 (35.5)	21 (32.8)	93 (34.8)	61 (38.1)	16 (34.0)	77 (37.2)
Any TEAE related to treatment	7 (3.4)	0	7 (2.6)	6 (3.8)	0	6 (2.9)
Any TEAE of special interest ^a^	7 (3.4)	1 (1.6)	8 (3.0)	6 (3.8)	1 (2.1)	7 (3.4)
Any TEAE of special interest ^a^ related to treatment	3 (1.5)	0	3 (1.1)	2 (1.3)	0	2 (1.0)
Any serious TEAE	13 (6.4)	6 (9.4)	19 (7.1)	11 (6.9)	4 (8.5)	15 (7.2)
Any serious TEAE related to treatment	0	0	0	0	0	0
Any TEAE leading to discontinuation	2 (1.0)	5 (7.8)	7 (2.6)	2 (1.3)	3 (6.4)	5 (2.4)
Any TEAE leading to discontinuation related to treatment	1 (0.5)	0	1 (0.4)	1 (0.6)	0	1 (0.5)
Any fatal TEAE	0	2 (3.1)	2 (0.7)	0	1 (2.1)	1 (0.5)
Any fatal TEAE related to treatment	0	0	0	0	0	0

^a^ Predefined TEAEs of special interest included all signs and symptoms that the investigator considered could indicate toxin spread. These could include dyspnea, respiratory tract infections, dry mouth, dysphagia, speech problems, diplopia, facial weakness, and general body weakness. INCO: incobotulinumtoxinA; PBO: placebo; TEAE: treatment-emergent adverse events; ULS: upper limb spasticity.

**Table 4:** Pooled safety data for incobotulinumtoxinA and placebo in older patients (aged ≥65 years) with upper limb spasticity by age group.

Number (%) of Patients with:	Age 65 to <75 Years			Age ≥ 75 Years		
	INCO (n = 172)	PBO (n = 55)	Total (n = 227)	INCO (n = 31)	PBO (n = 9)	Total (n = 40)
Any TEAE	60 (34.9)	19 (34.5)	79 (34.8)	12 (38.7)	2 (22.2)	14 (35.0)
Any TEAE related to treatment	6 (3.5)	0	6 (2.6)	1 (3.2)	0	1 (2.5)
Any TEAE of special interest ^a^	6 (3.5)	1 (1.8)	7 (3.1)	1 (3.2)	0	1 (2.5)
Any TEAE of special interest ^a^ related to treatment	2 (1.2)	0	2 (0.9)	1 (3.2)	0	1 (2.5)
Any serious TEAE	9 (5.2)	4 (7.3)	13 (5.7)	4 (12.9)	2 (22.2)	6 (15.0)
Any serious TEAE related to treatment	0	0	0	0	0	0
Any TEAE leading to discontinuation	1 (0.6)	4 (7.3)	5 (2.2)	1 (3.2)	1 (11.1)	2 (5.0)
Any TEAE leading to discontinuation related to treatment	0	0	0	1 (3.2)	0	1 (2.5)
Any fatal TEAE	0	1 (1.8)	1 (0.4)	0	1 (11.1)	1 (2.5)
Any fatal TEAE related to treatment	0	0	0	0	0	0

^a^ Predefined TEAEs of special interest included all signs and symptoms that the investigator considered could indicate toxin spread. These could include dyspnea, respiratory tract infections, dry mouth, dysphagia, speech problems, diplopia, facial weakness and general body weakness. INCO: incobotulinumtoxinA; PBO: placebo; TEAE: treatment-emergent adverse events.

**Table 5:** Baseline characteristics of older patients (aged ≥65 years) with moderate-to-severe upper limb spasticity who received a single dose of incobotulinumtoxinA or placebo.

Parameter	IncobotulinumtoxinA (n = 160)	Placebo (n = 47)	Total (n = 207)
Mean (SD) age, years	70.3 (4.2)	69.3 (3.5)	70.0 (4.1)
Age ≥ 75 years, n (%)	24 (15.0)	4 (8.5)	28 (13.5)
Female, n (%)	52 (32.5)	24 (51.1)	76 (36.7)
Race, n (%)			
White	117 (73.1)	34 (72.3)	151 (73.0)
Black or African American	1 (0.6)	1 (2.1)	2 (1.0)
Asian	30 (18.8)	12 (25.5)	42 (20.3)
Other/missing	12 (7.5)	0	12 (5.8)
Mean (SD) weight, kg	73.7 (13.9)	71.2 (13.9)	73.1 (13.9)
Baseline disease severity, n (%)			
Moderate	138 (86.3)	39 (83.0)	177 (85.5)
Severe	22 (13.8)	8 (17.0)	30 (14.5)
Mean (SD) time since spasticity diagnosis, years	5.5 (5.4)	4.7 (4.8)	5.3 (5.3)
Etiology of spasticity, n (%)			
Stroke—ischemic	91 (56.9)	31 (66.0)	122 (58.9)
Stroke—hemorrhagic	24 (15.0)	14 (29.8)	38 (18.4)
Stroke—not known	43 (26.9)	2 (4.3)	45 (21.7)
Other	2 (1.3)	0	2 (1.0)
Any concomitant medication, n (%)	159 (99.4)	47 (100.0)	206 (99.5)

SD: standard deviation.

## Data Availability

The data presented in this study are available on request from the corresponding author.

## Abbreviations

The following abbreviations are used in this manuscript:

*AESI*	adverse events of special interest
*AS*	Ashworth Scale
*mAS*	modified Ashworth Scale
*SD*	standard deviation
*ULS*	upper-limb spasticity
